# Links between COVID-19 and Alzheimer’s Disease—What Do We Already Know?

**DOI:** 10.3390/ijerph20032146

**Published:** 2023-01-25

**Authors:** Ewa Rudnicka-Drożak, Paulina Drożak, Grzegorz Mizerski, Tomasz Zaborowski, Barbara Ślusarska, Grzegorz Nowicki, Martyna Drożak

**Affiliations:** 1 Chair and Department of Family Medicine, Medical University of Lublin, Langiewicza 6a, 20-035 Lublin, Poland; 2 Student Scientific Society, Chair and Department of Family Medicine, Medical University of Lublin, Langiewicza 6a, 20-035 Lublin, Poland; 3 Department of Family and Geriatric Nursing, Faculty of Health Sciences, Medical University of Lublin, 20-081 Lublin, Poland

**Keywords:** Alzheimer’s disease, COVID-19, SARS-CoV-2, neuroinflammation

## Abstract

Alzheimer’s disease (AD) is a life-changing condition whose etiology is explained by several hypotheses. Recently, a new virus contributed to the evidence of viral involvement in AD: the severe acute respiratory syndrome coronavirus 2 (SARS-CoV-2), which causes the COVID-19 coronavirus disease. AD was found to be one of the most common COVID-19 comorbidities, and it was found to increase mortality from this disease as well. Moreover, AD patients were observed to present with the distinct clinical features of COVID-19, with delirium being prevalent in this group. The SARS-CoV-2 virus enters host cells through the angiotensin-converting enzyme 2 (ACE2) receptor. ACE2 is overexpressed in brains with AD, which thus increases the viral invasion. Furthermore, the inhibition of the ACE2 receptor by the SARS-CoV-2 virus may also decrease the brain-derived neurotrophic factor (BDNF), contributing to neurodegeneration. The ApoE ε4 allele, which increases the risk of AD, was found to facilitate the SARS-CoV-2 entry into cells. Furthermore, the neuroinflammation and oxidative stress existing in AD patients enhance the inflammatory response associated with COVID-19. Moreover, pandemic and associated social distancing measures negatively affected the mental health, cognitive function, and neuro-psychiatric symptoms of AD patients. This review comprehensively covers the links between COVID-19 and Alzheimer’s disease, including clinical presentation, molecular mechanisms, and the effects of social distancing.

## 1. Introduction

Alzheimer’s disease (AD) is a life-altering condition and a rapidly growing major public health concern. Currently, it affects around 44 million people globally; however, the number of patients is expected to double by 2050 due to the aging of the population. AD is also the fifth most common cause of mortality and a leading subtype of dementia, accounting for 60–80% of all dementia cases [1,2]. Today, AD is considered an incurable disease, though it may be prevented with several lifestyle strategies, such as implementing a healthy diet, exercise, socialization, and exposing oneself to mental challenges [3]. A definitive diagnosis of AD can only be confirmed through a postmortem analysis of brain tissue. However, positron emission tomography (PET) and cerebrospinal fluid (CSF) biomarkers, together with several clinical criteria, could facilitate diagnoses in living patients [4]. Nevertheless, despite the presence of certain diagnostic criteria of AD, an analysis of diagnosed dementia subtypes among over 3.1 million Medicare fee-for-service beneficiaries revealed that the most common diagnosis was dementia not otherwise specified, which was present in 46.1% of the beneficiaries. A diagnosis of AD/dementia not otherwise specified was present in 29.0% of the beneficiaries, and AD was diagnosed in 4.5% of this population [5].

Several hypotheses have been proposed regarding the etiology of AD. These include: a pathological deposit of amyloid beta (Aβ) in the extracellular spaces of neurons, formation of neurofibrillary tangles of hyperphosphorylated tau proteins inside neurons, inflammation, oxidative stress, cholinergic neuron damage, etc. [6]. Moreover, AD has been associated multiple times with viral etiology. The viral hypothesis of AD states that an exposure to viruses, such as Herpes Simplex type 1 and 2, Epstein–Barr virus, human cytomegalovirus, influenza virus, and hepatitis C virus, increases the risk of AD and plays an important role in the cognitive decline associated with this disease [7]. This hypothesis was formulated based on an observation that certain viral pathogens are found more commonly among Alzheimer’s patients [8]. Two main pathways have been proposed on how the viruses are involved in the AD pathology. The first is a direct pathway in which microbes directly infect the brain and promote the accumulation of Aβ and hyperphosphorylation of tau. The second is an indirect pathway, which results from the inflammatory effects of an infection [9]. Recently, a new virus contributed to the evidence of viral involvement in Alzheimer’s disease: the severe acute respiratory syndrome coronavirus 2 (SARS-CoV-2), which causes the COVID-19 coronavirus disease [10].

Evidence suggests that SARS-CoV-2 exhibits neurotropic properties and is able to invade the central nervous system (CNS). On a molecular level, the SARS-CoV-2 virus enters the cells via the binding of the spike protein (located on the surface of the virus) to the angiotensin-converting enzyme 2 (ACE2) receptor [11]. The expression of ACE2 receptors on endothelial cells, which compose the blood-brain barrier (BBB), mediates the SARS-CoV-2 invasion into the CNS [12]. At the organism level, the virus can reach CNS through several described routes. The first proposed mechanism is a hematogenous spread through infected leukocytes, which migrate to the brain [13]. Another way is a direct viral penetration from vascular endothelial cells through the BBB to the glial cells and then a transsynaptic transfer through the infected neurons. SARS-CoV-2 is also described as reaching CNS from the nasopharynx via retrograde axonal transport through the olfactory nerve to the olfactory bulb [12]. Moreover, neurological manifestations are common in a SARS-CoV-2 infection. Different studies demonstrated that from one-third to 60.8% of patients with this disease developed at least one neurological symptom, and such symptoms were more prevalent in patients with severe infections [14,15,16]. COVID-19 was also observed to be linked with neurodegenerative characteristics [17]. Patients with Parkinson’s disease (PD) were observed to have worsened motor and non-motor symptoms after being infected with SARS-CoV-2 [18]. A few case reports reported the development of AD, acute parkinsonism, or amyotrophic lateral sclerosis (ALS) after COVID-19 [19,20,21]. An analysis that included data concerning COVID-19 (severity, susceptibility, and hospitalization) and six major neurodegenerative disorders (AD, Lewy body dementia, frontotemporal dementia, PD, ALS, and multiple sclerosis) revealed that COVID-19 could increase the risk of AD. Notably, such causal association was not identified between COVID-19 and other neurodegenerative disorders included in this study [22]. Thus, among neurodegenerative diseases, AD appears to be particularly linked with SARS-CoV-2 infection.

This review comprehensively covers links between COVID-19 and Alzheimer’s disease, including the clinical presentation of SARS-CoV-2 infection in AD patients, potential underlying molecular mechanisms and effects of the pandemic-associated lockdown measures on mental health, neuropsychiatric symptoms, and cognitive decline of individuals with AD.

## 2. COVID-19 and Dementia: A Bidirectional Risk

It has been demonstrated that dementia increases the risk of COVID-19. A study conducted among the UK Biobank community cohort demonstrated that pre-existing dementia was a prominent risk factor [odds ratio (OR) = 3.07; 95% confidence interval (CI): 1.71 to 5.50] for severe SARS-CoV-2 infection. This risk factor was stronger than chronic obstructive pulmonary disease (COPD), type 2 diabetes, and depression [23]. Research carried out in the United States showed that increased risk for SARS-CoV-2 infection was strongest for vascular dementia, followed by presenile dementia, AD, senile dementia, and post-traumatic dementia. Moreover, the risk of COVID-19 among individuals with dementia was higher among Black people than White people [24]. Another study indicated that among 16749 people hospitalized in the UK due to COVID-19, dementia was one of the most common comorbidities [25]. Moreover, one meta-analysis of 10 studies indicated that the mortality rate of COVID-19 infection among people with dementia was higher than that for those without dementia (OR = 5.17; 95% CI: 2.31 to 11.59) [26]. A study from Spain indicated that Alzheimer’s disease was the most common diagnosis of cognitive impairment among patients who died from COVID-19 [27]. Furthermore, COVID-19 survivors were found to be at a higher risk of a new-onset dementia diagnosis in a 6-month follow-up after an infection, compared to individuals from the control group. In one study, the incidence of dementia among SARS-CoV-2 infection survivors was 1.39 times higher than that among those in the control group [28].

## 3. Different Clinical Presentation of COVID-19 among Dementia Patients

A SARS-CoV-2 infection among individuals with dementia is characterized by a specific clinical presentation, with delirium being a leading symptom and occurring in 36.2% of cases, compared to the prevalence of 11.6% in the dementia-free control group. Furthermore, dementia patients less often presented with other COVID-19 symptoms, such as dyspnea, myalgias, chills, nausea or vomiting, and headache, in comparison to patients from the control group (*p* < 0.05). The mortality rate at 1 month was higher among the patients in the group with dementia than among the dementia-free individuals (50.0% vs. 35.4%; *p* = 0.006) [29]. One study indicated that at the onset of COVID-19, delirium and confusion appeared in 82.4% of patients with dementia, among whom the most common cause of dementia was AD. Other frequent COVID-19 onset symptoms included first asthenia (76.8%) and fever (72.8%) and later polypnea (51.2%) and desaturation (50.4%). Moreover, falls occurred among 35.2% of patients during the initial phase of the disease. Persistent confusion and behavioral disorders were present among 19.2% of survivors. CRP and chronic kidney disease at admission were found to be independent risk factors of death [30]. In another study, the most frequent onset symptom of SARS-CoV-2 infection was delirium, particularly in the hypoactive form, and a decline in functional status. In the same study, the mortality rate was higher among patients with dementia compared to subjects without dementia (62.2% vs. 26.2%; *p* < 0.001) [31].

## 4. Angiotensin-Converting Enzyme 2 (ACE2) Receptor

Angiotensin-converting enzyme 2 (ACE2) is a homolog of angiotensin-converting enzyme (ACE) and catalyzes a hydrolysis of the vasoconstrictive Angiotensin II (Ang II) into Angiotensin-(1-7) [Ang-(1-7)], which acts as a vasodilator [32]. ACE2, together with Ang-(1-7) and the receptor Mas, create an ACE2/Ang-(1-7)/Mas axis, which counteracts the actions of an ACE/Ang II/AT1R axis in the brain and peripheral organs. These two axes, which counterbalance each other, are a part of the renin angiotensin system (RAS) and play an important role in the regulation of blood pressure [33,34]. Moreover, research points to a crucial role of the ACE2/Ang-(1-7)/Mas axis in sustaining proper cognitive function and protecting against neurodegeneration, contrary to ACE and Ang II, which were observed to induce cognitive decline [35,36,37,38]. It has been demonstrated that Ang II type 1 (AT1) receptor blockers (ARBs) are associated with a significantly reduced risk of incidence and progression of dementia and AD compared to ACE inhibitors (ACEI) and other cardiovascular drugs [39]. Another study found that the level of Ang-(1-7) was significantly reduced in the brains of sporadic AD model mice. Moreover, the same study reported that the Ang-(1-7) level in the cerebral cortex and hippocampus was inversely correlated with tau hyperphosphorylation [40].

ACE2 has been found to be expressed all over the human body, with the highest expression in the small intestine, kidneys, heart, testis, thyroid, and adipose tissue; the lowest expression levels are in the blood, bone marrow, blood vessels, brain, spleen, and muscles. A medium expression level of ACE2 has been detected in the lungs, liver, colon, bladder, and adrenal gland [41]. ACE2 occurs in two forms: membrane-bound ACE2 (mACE2), also referred to as the ACE2 receptor, and soluble ACE2 (sACE2), which is located in the plasma [42]. The SARS-CoV-2 virus enters the cells through an interaction between the viral S1 spike protein and mACE2, as ACE2 lacks the crucial membrane machinery that would facilitate cell invasion [43,44,45].

A recent study found that AD patients showed reduced serum ACE2 activity compared to patients in healthy control groups [46]. However, another recent study reported an upregulated protein expression level of ACE2 in the brains of AD patients, which was independent of age, gender, and severity of disease; namely, a significant upregulation of ACE2 was observed even in patients with mild AD [47].

Aβ43 and Aβ42, longer forms of Aβ, are the main contributors to the Aβ accumulation in the brain during the course of AD due to their neurotoxicity and high amyloidogenicity [48]. ACE2 has been found to convert Aβ43 to Aβ42, which is then converted by ACE to the less toxic Aβ40, which may have neuroprotective properties. This inhibits amyloid aggregation of Aβ42 in in vivo studies and slows down amyloid deposition of Aβ42 in in vitro studies [49,50,51].

### Overlaps between ACE2 and COVID-19

Aβ42 was demonstrated to be able to bind with numerous viral proteins, with an especially high affinity for the spike protein S1 subunit of SARS-CoV-2 and ACE2. Moreover, in the SARS-CoV-2 pseudovirus infection model, Aβ42 strengthened the binding of the viral spike protein to ACE2, elevated viral entry, and increased IL-6 production. Such action was not observed in the case of Aβ40 [52]. Thus, the already present Aβ pathology in a brain with AD may facilitate the SARS-CoV-2 viral entry into brain cells; moreover, it may increase the production of proinflammatory cytokines.

The hippocampus is a crucial brain structure affected in AD [53]. Research has suggested that an enhancement of ACE2 activity in mouse models of AD lowers hippocampal Aβ deposition, reduces levels of Aβ42, hyperphosphorylated tau and inflammatory cytokines in the brain, and restores a cognitive deficit [54,55,56]. A recent study reported higher ACE2 protein expression levels in the hippocampal tissues of individuals with AD, compared to healthy individuals in the control group [47]. Another study also reported significantly upregulated ACE2 expression in the CA1 region of the hippocampus as well as the temporal lobe and occipital lobe. Since the temporal lobe and hippocampus are regions particularly involved in the neuroinflammatory pathology during the course of AD, the results of this study suggest a significant overlap between COVID-19 and AD [57]. Furthermore, these findings indicate that an infection of AD patients with SARS-CoV-2 leads to increased viral entry into the brain cells of such individuals, compared to healthy individuals, due to increased ACE2 expression in AD.

**Figure 1:**
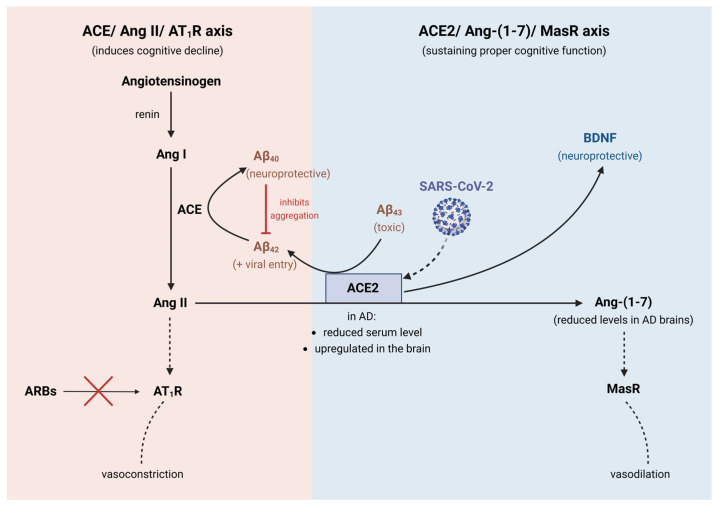
A schematic representation of ACE/Ang II/AT1R and ACE2/Ang-(1-7)/Mas axes in AD and SARS-CoV-2 invasion with ACE2 receptor in the spotlight. The ACE2 receptor, through which SARS-CoV-2 enters cells, is upregulated in the brain with AD, which increases viral entry. Moreover, ACE2 transforms Aβ_43_ into Aβ_42_, which additionally intensifies the invasion of this virus. Solid arrows represent a transformation of one compound into other, and dotted arrows represent a ligand binding to the receptor. The dotted lines represent an effect caused by activation of the receptor and red lines represent inhibition. Abbreviations: Aβ—amyloid beta; ACE—angiotensin converting enzyme; AD—Alzheimer’s disease; Ang—angiotensin; ARBs—angiotensin receptor blockers; AT_1_R—angiotensin II type 1 receptor; BDNF—brain-derived neurotrophic factor; MasR – Mas receptor.

ACE2 is one of the main enzymes that regulate the release of important neurotrophic factors, such as brain-derived neurotrophic factor (BDNF) [58]. BDNF has a crucial function in neurogenesis, neurodevelopment, cognition, and the prevention of neurodegeneration [59]. A study involving ACE2 knockout mice showed that a deficiency of this enzyme led to an impairment of cognitive function, which was suggested to be due to a decrease in BDNF and elevated oxidative stress [38]. A hypothesis has thus been proposed that SARS-CoV-2 infection, through causing an inhibition of ACE2 and hence BDNF, evokes neurodegenerative changes through an increase in neuroinflammation, oxidative stress, and apoptosis [58]. Figure 1. highlights the role of an ACE2 receptor in an AD patient with SARS-CoV-2.

## 5. Apolipoprotein E (ApoE)

Apolipoprotein E (ApoE) is a protein whose main function is to transport cholesterol and other lipids to neuronal cells [60]. It is mainly produced in astrocytes and microglial cells, and, under certain conditions, also in neurons [61]. ApoE occurs in three isoforms: apoE2, apoE3, and apoE4, which are encoded by three alleles: ε2, ε3, and ε4, respectively. These isoforms differ among each other regarding certain functional properties concerning lipid transportation or neuronal plasticity [60,62]. A study has found that apoE2 and apoE3, but not apoE4, inhibit Aβ aggregation and neurotoxicity [63]. Thus, individuals possessing the ε4 allele of ApoE were found to be at the highest risk of sporadic AD [64].

### Overlaps between ApoE and COVID-19

Analysis of the data from the UK Biobank community cohort indicated that ApoE ε4ε4 homozygotes were 2.31 times more likely to test positive for COVID-19 than ε3ε3 homozygotes. Moreover, the ApoE ε4ε4 allele was found to increase the risk of severe COVID-19, independent of pre-existing dementia, type 2 diabetes, and cardiovascular disease [65].

A study performed using the human-induced pluripotent stem cells (hiPSCs) observed a higher SARS-CoV-2 infection rate among those with ApoE4/4 isogenic neurons and astrocytes compared to those with the ApoE3/3 genotype. Furthermore, upon SARS-CoV-2 infection, ApoE4/4 astrocytes showed increased fragmentation of the nucleus and a larger size, which are apoptotic markers [66]. In another study, high blood levels of cholesterol were demonstrated to facilitate the SARS-CoV-2 endocytic entry into cells through ACE2 receptors by binding cholesterol to the ApoE receptors [67]. Previous research has indicated that ApoE facilitates or participates in the entry into cells of other viruses, such as the hepatitis C virus (HCV), herpes simplex virus (HSV), or human immunodeficiency virus (HIV) [68,69]. These findings suggest a higher susceptibility of ApoE ε4 neurons and astrocytes to SARS-CoV-2 infection and its subsequent increased severity.

## 6. Neuroinflammation in AD

During the past few decades, the inflammatory hypothesis of AD has emerged as one of the three core pathologies of this disease, alongside Aβ accumulation and presence of neurofibrillary tangles. Behind this hypothesis are findings that continuous activation of microglia (macrophages resident in the brain) and immune cells aggravates both Aβ and tau pathology. Moreover, such activation could provide a link between other conditions and AD pathogenesis [70]. A brain with AD is characterized by the presence of chronic inflammatory conditions. The activated microglial cells secrete a wide variety of proinflammatory cytokines, such as interleukin-6 (IL-6), interleukin-1β (IL-1β), and tumor necrosis factor α (TNF-α), which were found to be elevated in the tissue of a brain with AD in a postmortem study [71]. Furthermore, AD patients were found to have higher IL-6 and TNF-α serum levels, compared to healthy individuals [72]. An increased peripheral level of IL-6 during late midlife was demonstrated to predict cognitive decline in a 10-year observation study [73].

### 6.1. Cytokine Storm and NLRP3 Inflammasome

Cytokine storm, a state of hyperinflammation, is a hallmark of severe SARS-CoV-2 infection [74]. Multiple studies have demonstrated elevated levels during COVID-19 of numerous proinflammatory cytokines, such as IL-1β, interleukin-2 (IL-2), IL-6, interleukin-10 (IL-10), TNF-α, interferon (IFN) γ, IFN-γ-inducible protein 10 (IP-10), monocyte chemoattractant protein-1 (MCP-1), and granulocyte macrophage-colony stimulating factor (GM-CSF). Moreover, the above-mentioned proinflammatory cytokines were found to be positively correlated with the severity of the disease [75,76,77,78,79,80,81,82,83,84,85,86,87,88,89]. This dysregulated immune response, which results in heightened levels of cytokines, may be partly due to an activation of the NLR family pyrin domain-containing protein 3 (NLRP3) inflammasome by open reading frame 3a (ORF3a), an accessory protein of the SARS-CoV-2 virus [80,81,82,83]. Studies have demonstrated that Aβ plaques and tau aggregates stimulate an activation of the microglial NLRP3 inflammasome [84,85]. Activation of the NLRP3 inflammasome subsequently impairs normal microglial function, which leads to reduced clearance of Aβ42 in the brain [86]. Activation of the NLRP3 inflammasome has been described as a key neuroinflammatory pathway in AD that leads to cognitive decline [87]. Since the ORF3a protein of the SARS-CoV-2 virus is capable of activating the NLRP3 inflammasome, this mechanism could further amplify the already present neuroinflammation caused by the activation of this inflammasome during the course of AD.

### 6.2. Apolipoprotein E

It was reported that humans possessing the ApoE ε3ε4 genotype exhibited higher plasma levels of IL-6 and TNF-α and higher hyperthermia compared to individuals with the ApoE ε3ε3 genotype following an intravenous injection of lipopolysaccharide [88]. Thus, it is possible that the ApoE ε4 allele could exacerbate the immune response caused by SARS-CoV-2 by upregulating the inflammatory pathways.

### 6.3. Acetylcholine

Acetylcholine (Ach) is an excitatory neurotransmitter present in the CNS that is synthesized from acetyl-CoA and choline by an enzyme choline acetyltransferase (ChAT) and hydrolyzed by an enzyme acetylcholinesterase (AChE) [82]. Ach plays an important role in cognitive and memory functions [90]. Ach was also found to mitigate the release of several proinflammatory cytokines, such as IL-1β, IL6, IL-18, and TNF-α, following a lipopolysaccharide stimulation of human macrophage cultures [91]. The cholinergic hypothesis of AD states that AD symptoms develop due to a decline in cholinergic transmission, which is caused by the death of Ach-producing neurons, loss of Ach receptors, alterations in cholinergic synapses, and accumulation of AChE [92]. Additionally, cortical ChAT activity was found to be inversely correlated with AD disease severity [93]. Thus, ACEI, such as galantamine, rivastigmine, and donepezil, are among the currently implemented effective therapeutic strategies for the treatment of AD [94]. A very recent clinical trial revealed that pirydostygmine, another AChEI, reduces mortality among patients hospitalized due to severe COVID-19. The 90-day mortality in the pirydostygmine group equaled 9.5%, compared to 20.2% in the placebo group, and this difference was statistically significant (HR 0.43, CI 0.2–0.93, *p*  =  0.03) [95]. Another interesting study revealed that a high prenatal level of choline in a mother’s body can prevent fetal brain development from adverse effects of a SARS-CoV-2 infection [96].

### 6.4. The Oxidative Stress Hypothesis of AD

Oxidative stress was found to be another important factor contributing to the initiation and progression of AD. Oxidative stress is a result of redox imbalance in a situation when an excessive production of reactive oxygen species (ROS) takes place. This leads, as a consequence, to the loss of neurons [97]. Oxidative stress was found to facilitate the accumulation of Aβ in research conducted on AD mice [98,99]. In turn, numerous studies, also carried out on AD model mice, revealed that Aβ promotes oxidative stress [100,101,102,103,104]. A large amount of evidence has also suggested an involvement of oxidative stress in hyperphosphorylation and polymerization of tau [105,106,107,108,109]. Furthermore, multiple studies have pointed out that oxidative stress increases with age, and AD largely affects elderly individuals [110,111,112].

In SARS-CoV-2 infection, oxidative stress was found to play a role in the perpetuation of the cytokine storm and increased cellular hypoxia [113,114]. As a response to SARS-CoV-2 infection, ROS are overproduced, since they are a part of the toxic innate immune response against viral agents [115]. The addition of the oxidative stress caused by ROS overproduction during a SARS-CoV-2 infection to the already increased oxidative stress in AD patients due to age is a potential mechanism for COVID-19 to exacerbate AD [116].

## 7. Mental Health of AD Patients during COVID-19 Pandemic and Associated Social Distancing Measures

The quarantine measures taken during the COVID-19 pandemic have left a mark on the mental health of the global population, which manifested as anxiety, distress, depression, and post-traumatic symptoms [117]. Research indicated that the lockdown was also not indifferent to AD patients. A study that included 58 individuals with a probable diagnosis of AD who were living in retirement homes across France reported an increase in depression and anxiety during the COVID-19 crisis compared to the time before [118]. Another study, also carried out among AD residents of retirement homes, obtained similar results and attributed them to a restriction of contact between patients and their family members, as well as a restriction of other activities [119]. A decline in the mood of AD patients was observed to further extend to a time after confinement [120]. Notably, a study involving cognitively unimpaired adults in the pre-clinical stage of AD showed that β-amyloid positivity, female gender, younger age, and lower education were associated with greater anxious-depressive symptoms during lockdown [121].

### 7.1. Neuropsychiatric Symptoms and Cognitive Decline

In one study, caregivers of AD patients described increased hallucinatory experiences by the patients during lockdown, compared to before lockdown [122]. Another study indicated that during the COVID-19 pandemic, AD patients experienced a decline in cognitive abilities, deterioration of neuropsychiatric symptoms, and more sleep disturbances than before the pandemic. However, interestingly, this same study found that the incidence of rapid cognitive decline (RCD) during the COVID-19 pandemic was lower among AD individuals (19.1%) compared to people in the AD control group, who were observed during the same time period before the pandemic (36.6%). Thus, AD patients were 0.41 times (95% CI: 0.23–0.72) less likely to experience RCD during the COVID-19 pandemic than before the pandemic. The authors suggested that although confinement may facilitate the cognitive and neuropsychiatric deterioration of AD, it may at the same time prevent RCD [123]. Another study reported that during confinement, 26.3% of AD patients demonstrated neuropsychiatric changes, and those patients had worse cognitive function, as measured by their MMSE score, compared to those who did not demonstrate neuropsychiatric changes. Moreover, the severity of these changes correlated positively with the duration of the confinement [124]. A similar study reported that 54.3% of AD patients demonstrated a decline in their MMSE score, and over 40% of them experienced a deterioration in Neuropsychiatric Inventory (NPI) in a 1-year follow-up study between October of 2019 and November of 2020. Furthermore, it was found that even 6 months after a strict lockdown, social isolation and physical inactivity correlated with the worsening of cognitive function and neuropsychiatric symptoms in AD patients [125].

### 7.2. Nursing Burden and Mental Health of AD Caregivers

The COVID-19 pandemic was also found to affect the nursing burden and mental health of AD patients’ caregivers. One study found that 36.6% of AD caregivers experienced an increased caregiver burden, whereas 31.7% of them experienced worsening anxiety and 29.6% of them experienced worsening depression [126]. However, another study revealed that a majority of AD caregivers (>60%) reported that family and friends, hobbies, and music helped them cope with negative feelings associated with the SARS-CoV-2 pandemic and lockdown. Moreover, receiving social support by caregivers was strongly associated with experiencing fewer negative emotions, such as loneliness, depression, anxiety, uncertainty, fatigue, and stress [127].

## 8. Conclusions

The SARS-CoV-2 virus, which caused the COVID-19 pandemic, highly affected individuals with Alzheimer’s disease in multiple ways. AD was found to be one of the most common COVID-19 comorbidities. A co-occurrence of AD with COVID-19 was also observed to significantly increase patients’ mortality. Furthermore, AD patients were found to have a distinctive clinical presentation of COVID-19, with delirium being a common symptom. Several pre-existing pathologies in patients with AD were also found to be involved in the SARS-CoV-2 infection, and thus an overlap between those two diseases has been identified. These areas of overlap concern: (1) the ACE2 receptor, through which SARS-CoV-2 enters cells and whose expression in AD is upregulated, which leads to increased viral entry; (2) the ApoE ε4 allele, which facilitates the SARS-CoV-2 viral entry into cells; and (3) an enhancement of inflammation and oxidative stress already present in AD by the SARS-CoV-2 infection. Furthermore, the COVID-19 pandemic and associated social distancing measures negatively affected mental health, cognitive function, and neuropsychiatric symptoms of AD patients. Given all the evidence, AD and COVID-19 appear to be linked with each other in multiple important ways.

### Future Directions

Since research suggests a relationship between Alzheimer’s disease and COVID-19, there is a need to develop and implement proper preventive strategies that would lower the risk of developing AD in people infected with SARS-CoV-2. A longitudinal observation of COVID-19 patients should be conducted in order to detect the long-term neurological consequences of a SARS-CoV-2 infection, as well as to identify symptoms of dementia as soon as they emerge. Large-scale retrospective studies, including medical records from the times before the pandemic, should be carried out in order to determine the risk factors for the development or aggravation of AD after COVID-19. However, above all, it appears particularly important to prevent AD patients from being infected with the SARS-CoV-2 virus in the first place.

## Data Availability

Not applicable.

## Abbreviations

ACE	angiotensin-converting enzyme
ACE2	angiotensin-converting enzyme 2
ACEI	angiotensin-converting enzyme inhibitors
AChE	acetylcholinesterase
AD	Alzheimer’s disease
ALS	amyotrophic lateral sclerosis
Ang II	angiotensin II
Ang-(1-7)	angiotensin-(1-7)
ApoE	apolipoprotein E
ARBs	Angiotensin II type 1 (AT1) receptor blockers
BBB	blood-brain barrier
BDNF	brain-derived neurotrophic factor
ChAT	choline acetyltransferase
CI	confidence interval
CNS	central nervous system
COPD	chronic obstructive pulmonary disease
COVID-19	coronavirus disease 2019
CSF	cerebrospinal fluid
GM-CSF	granulocyte macrophage-colony stimulating factor
HCV	hepatitis C virus
HiPSCs	human-induced pluripotent stem cells
IFN	interferon
IL-1β	interleukin-1β
IL-2	interleukin-2
IL-6	interleukin-6
IL-10	interleukin-10
IP-10	interferon-γ-inducible protein 10
HIV	human immunodeficiency virus
HSV	herpes simplex virus
mACE2	membrane-bound ACE2
MCP-1	monocyte chemoattractant protein-1
MMSE	Mini-Mental State Examination
NPI	Neuropsychiatric Inventory
PD	Parkinson’s disease
PET	positron emission tomography
NLRP3	NLR family pyrin domain-containing protein 3
OR	odds ratio
ORF3a	open reading frame 3a
RCD	rapid cognitive decline
ROS	reactive oxygen species
sACE2	soluble ACE2
SARS-CoV-2	severe acute respiratory syndrome coronavirus 2
TNF-α	tumor necrosis factor α
